# Inhaled and Systemic Steroids for Bronchopulmonary Dysplasia: Targeting Inflammation and Oxidative Stress

**DOI:** 10.3390/antiox14070869

**Published:** 2025-07-16

**Authors:** Francesca Galletta, Alessandra Li Pomi, Sara Manti, Eloisa Gitto

**Affiliations:** 1 Pediatric Unit, Department of Human Pathology in Adult and Developmental Age ‘Gaetano Barresi’, University of Messina, 98124 Messina, Italy; francygall.92@gmail.com; 2 Neonatal and Pediatric Intensive Care Unit, Department of Clinical and Experimental Medicine, University of Messina, 98122 Messina, Italy; alessandra.lipomi92@gmail.com (A.L.P.); egitto@unime.it (E.G.)

**Keywords:** bronchopulmonary dysplasia, systemic steroids, inhaled steroids, lung injury, inflammation, oxidative stress, newborn

## Abstract

Bronchopulmonary dysplasia (BPD) remains a significant complication of preterm birth, characterized by impaired alveolar and vascular development, chronic lung inflammation, and long-term respiratory morbidity. Corticosteroids, both systemic and inhaled, have been widely investigated as potential therapeutic agents due to their anti-inflammatory properties and their emerging role in modulating oxidative stress. This narrative review explores the current evidence regarding the use of inhaled and systemic corticosteroids in the prevention and management of BPD, analyzing their efficacy, safety profiles, and long-term outcomes. While systemic corticosteroids, particularly dexamethasone, have demonstrated benefits in reducing ventilator dependence and lung inflammation, concerns regarding adverse neurodevelopmental effects have limited their routine use. Inhaled steroids have been proposed as a safer alternative, but their role in altering the disease trajectory remains controversial. A better understanding of the optimal timing, dosage, and patient selection is essential to maximize benefits while minimizing risks. Future research should focus on optimizing dosing strategies and identifying subgroups of preterm infants who may derive the greatest benefit from corticosteroid therapy.

## 1. Introduction

Bronchopulmonary dysplasia (BPD) is a chronic lung disease primarily affecting preterm infants, particularly those born before 27 weeks of gestation [1]. Despite advances in neonatal care, its prevalence remains high, with up to 75% of extremely preterm infants diagnosed with the condition [2]. BPD is currently defined by the need for supplemental oxygen or respiratory support at 36 weeks’ postmenstrual age (PMA) and is categorized into three severity grades based on oxygen dependency and ventilation requirements [3]. Severe cases of BPD are associated with prolonged hospital stays, difficulties in weaning from respiratory support, feeding intolerance, and intermittent hypoxic episodes [4,5]. Many affected infants require home oxygen therapy upon discharge [5]. The long-term consequences of BPD extend into adolescence and adulthood, often leading to impaired lung function, recurrent respiratory infections, and neurodevelopmental challenges [6,7]. Furthermore, severe cases carry a significant mortality risk, with rates reaching 3% during the initial hospital stay and up to 20% within two years in infants requiring tracheostomy [8,9]. While initially described as a consequence of high oxygen exposure and prolonged mechanical ventilation, it is now understood as a multifactorial disease where immune dysregulation and abnormal lung development play critical roles [3].

### 1.1. The Role of Inflammation in the Pathogenesis of BPD

**Figure 1:**
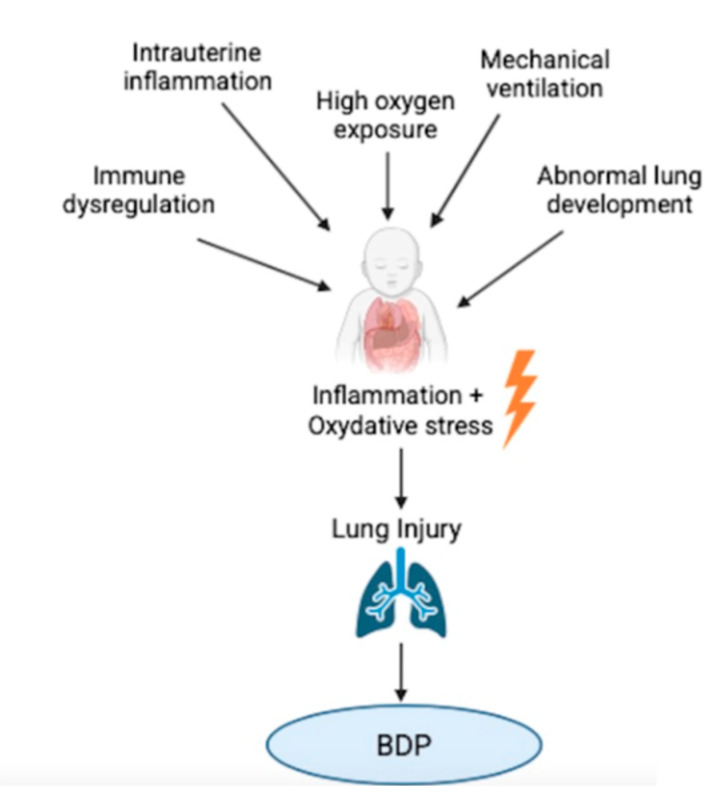
Conceptual model illustrating the proposed role of oxidative stress and inflammation in the pathogenesis of bronchopulmonary dysplasia (BPD) and the potential modulatory effects of corticosteroids. The diagram integrates current knowledge from experimental and clinical studies to depict how antenatal and postnatal factors trigger reactive oxygen species (ROS) production and pro-inflammatory cascades, contributing to lung injury and impaired alveolar development. Corticosteroids are shown to act at multiple levels, modulating inflammation and oxidative stress pathways.

Inflammation plays a central role in the pathogenesis of BPD [Figure 1]. Both prenatal and postnatal factors contribute to the development of an exaggerated and persistent inflammatory response in the immature lung [10,11]. Particularly, one of the main prenatal factor contributors to BPD is intrauterine inflammation, often associated with conditions such as chorioamnionitis, which can elicit a fetal inflammatory response. Prenatal exposure to inflammation may cause early immune activation, predisposing the neonatal lung to exaggerated inflammatory responses after birth [10]. This immune hyperactivation contributes to the infiltration of inflammatory cells and the release of pro-inflammatory cytokines, including IL-1β, IL-6, IL-8, and TNF-α, which recruit and activate neutrophils and macrophages. These cells, in turn, release reactive oxygen species (ROS) and proteases, exacerbating alveolar and vascular injury [12]. Postnatal exposures to oxygen therapy, mechanical ventilation, and infections further exacerbate the inflammatory state, promoting endothelial dysfunction, increased vascular permeability, and interstitial edema [11,13]. The persistence of inflammation disrupts the normal processes of lung development, including alveolarization and angiogenesis, leading to impaired gas exchange and chronic lung dysfunction [13]. Studies indicate that inflammatory signaling is mediated by pattern recognition receptors (PRRs), such as toll-like receptors (TLRs) and the receptor for advanced glycation end-products (RAGE), which respond to danger-associated molecular patterns (DAMPs) and pathogen-associated molecular patterns (PAMPs) [11]. Moreover, recent microbiome studies suggest that an altered lung microbiota composition in preterm infants may contribute to heightened inflammation, with an increased prevalence of potentially pathogenic species such as *Ureaplasma* spp. and *Corynebacterium* spp., and a reduction in beneficial Lactobacilli. This dysbiosis may further exacerbate immune dysregulation and predispose preterm infants to BPD [14].

### 1.2. The Role of Oxidative Stress in the Pathogenesis of BPD

Oxidative stress (OS) is a central contributor to the pathogenesis of BPD, intricately linked to both inflammation and impaired lung development [Figure 1]. Preterm infants are particularly susceptible due to their abrupt shift from a hypoxic intrauterine environment to a hyperoxic extrauterine setting, compounded by mechanical ventilation and oxygen supplementation [12]. This transition challenges the immature antioxidant defense systems of the preterm lung, resulting in the excessive generation of ROS such as superoxide anions, hydrogen peroxide, and hydroxyl radicals. These ROS induce lipid peroxidation, protein oxidation, and DNA damage, leading to epithelial and endothelial cell injury, increased alveolar–capillary permeability, and interstitial edema [11]. Emerging evidence highlights a dynamic interplay between oxidative stress and inflammation, with ROS capable of activating transcription factors such as NF-κB and AP-1, further amplifying inflammatory response and perpetuating lung injury [11]. Furthermore, OS has been shown to modulate the expression of genes crucial for lung development, including peroxisome proliferator-activated receptor gamma (PPARγ) and β-catenin. The hyperoxia-induced downregulation of PPARγ impairs alveolar epithelial cell growth and differentiation, while persistent β-catenin activation is associated with abnormal extracellular matrix deposition and fibrosis [11]. Experimental models have demonstrated that the pharmacological modulation of these pathways can mitigate hyperoxia-induced lung injury [14]. Metabolomic and proteomic studies have identified specific oxidative stress biomarkers, such as lipid peroxidation products and oxidized proteins, in the early postnatal period of infants who subsequently developed BPD. These findings suggest that OS may begin antenatally, with oxidative damage markers detectable shortly after birth, and that early antioxidant strategies might be necessary for effective prevention [14]. Despite the role of these biomarkers in BPD onset and progression, their regulation in response to corticosteroid treatment has yet to be systematically characterized in clinical populations. While preclinical studies indicate that corticosteroids may influence oxidative stress mechanisms and related proteomic pathways, clinical investigations demonstrating the direct associations between steroid therapy and the shifts in metabolomic or proteomic profiles in neonates remain limited [14]. Further research is necessary to determine whether these biomarkers could serve as reliable tools to predict or monitor therapeutic response, ultimately supporting the development of more personalized corticosteroid-based interventions in the management of BPD [14].

### 1.3. Rationale for the Use of Steroids in the Management of BPD

Therapeutic strategies have focused on modulating inflammatory and oxidative stress responses to mitigate lung injury and promote normal pulmonary development. Among these, glucocorticoids have been considered for their ability to suppress inflammation and stabilize lung function [4]. Additionally, corticosteroids may provide a protective effect by not only dampening inflammation but also indirectly modulating oxidative stress. Their anti-inflammatory action includes the suppression of pro-oxidant cytokine release and the promotion of antioxidant pathways, potentially reducing ROS production and its downstream effects [15,16]. Some studies have shown that steroid administration is associated with decreased levels of oxidative stress biomarkers, suggesting a synergistic role between anti-inflammatory and antioxidant effects [15,16]. However, their use remains controversial, necessitating a careful evaluation of their benefits and potential risks. This narrative review aims to explore the role of corticosteroids as potential therapeutic approaches targeting lung inflammation and OS. We performed a literature search using the main scientific databases, including PubMed, Scopus, Embase, and Google Scholar, selecting only relevant articles published in high-quality journals and written in English to ensure the reliability and scientific rigor of the review.

## 2. Systemic Steroids in BPD

The primary corticosteroids used for their anti-inflammatory effects in infants with BPD include dexamethasone, hydrocortisone, prednisolone, and methylprednisolone [17]. Although other corticosteroids like betamethasone and fludrocortisone have been considered, there is insufficient and inconsistent data supporting their use in evolving BPD, and they will not be further reviewed here [17].

### 2.1. Dexamethasone in BPD

Dexamethasone is among the most extensively studied corticosteroids in preterm neonates, particularly for its role in reducing BPD. Several randomized controlled trials (RCTs) have shown that early systemic dexamethasone administration, particularly within the first 7 days of life, reduced the incidence of BPD at 36 weeks PMA (RR 0.72, 95% CI 0.63–0.82; 17 studies, 2791 infants; high-certainty evidence), as well as the combined outcome of death or BPD (RR 0.88, 95% CI 0.81–0.95; 17 studies, 2791 infants; high-certainty evidence) [18,19,20]. However, the effect on mortality alone was not significant (RR 0.95, 95% CI 0.85–1.06; 31 studies, 4373 infants; high-certainty evidence), and the certainty of the evidence for BPD across all studies was downgraded to moderate due to suspected publication bias [18]. Moreover, dexamethasone facilitates extubation, with significant improvements noted as early as day 3 and sustained through day 10 [18]. Late administration (after seven days of age) has also shown consistent benefits, including a reduced need for rescue corticosteroids, lower extubation failure rates, and decreased home oxygen dependency [17,18]. Regarding the dosage used, the remote Dexamethasone: A Randomized Trial (DART) protocol used a cumulative dose of 0.89 mg/kg over 10 days in extremely low birth-weight infants, achieving a significant facilitation of extubation without increasing mortality or neurodevelopmental impairment [19]. However, more recent systematic reviews and meta-analyses indicated that moderately early initiation (between 8 and 14 days) with medium cumulative doses (2–4 mg/kg) appeared to provide the most favorable outcomes in preventing BPD, with up to a 57% relative risk reduction in some studies [20,21]. The Canadian Paediatric Society endorses the initiation of dexamethasone at 0.15–0.2 mg/kg/day for 7–10 days in neonates requiring mechanical ventilation beyond the first postnatal week [22]. This regimen aligns with evidence suggesting improved pulmonary outcomes when therapy begins between 8 and 14 days of age and maintains a cumulative dose between 2 and 4 mg/kg. In summary, longer treatment durations and moderate dosing regimens appear to be more effective than shorter or low-dose approaches [19,21]. However, despite these pulmonary benefits, dexamethasone therapy carries notable risks, particularly when initiated early or at higher doses. Its early use has been consistently linked with an increased risk of cerebral palsy (CP) and neuromotor impairment, even with courses as brief as three days [20]. Doyle et al. [23] found that early administration of dexamethasone raised the incidence of CP, while late administration did not. Zeng et al. reported that high-dose early regimens (>3 mg/kg) heightened this risk further [24]. Gastrointestinal complications are also a concern. The early administration of dexamethasone, especially in conjunction with indomethacin, has been associated with gastrointestinal perforation [25]. Other adverse events include hyperglycemia, hypertension, and, as recently reported, hypoglycemia, a less commonly recognized effect of dexamethasone in neonates, warranting closer glucose monitoring during treatment [23]. Infections remain a common complication, with some studies reporting incidences exceeding 50% [23,26]. Nevertheless, neither dexamethasone nor other systemic steroids, such as hydrocortisone, have been shown to increase the risk of necrotizing enterocolitis (NEC), retinopathy of prematurity (ROP), or sepsis in a consistent manner across studies [26]. Ultimately, the risk–benefit profile of dexamethasone is highly dependent on timing, dosage, and duration. While early administration may reduce BPD incidence, it increases the likelihood of neurodevelopmental harm. Conversely, delayed and moderately dosed regimens appear to preserve pulmonary efficacy while minimizing long-term adverse outcomes. In current clinical practices, dexamethasone is generally discouraged during the first week of life [27]. Its administration beyond this period should be limited to preterm infants who are at a high risk of developing BPD. One approach to identifying such high-risk neonates is using predictive tools, such as the neonatal BPD Outcome Estimator [28].

### 2.2. Hydrocortisone in BPD

Hydrocortisone, a corticosteroid with predominant mineralocorticoid activity, has also been widely investigated for its potential role in preventing BPD in preterm infants [17]. Emerging evidence suggests that early administration may offer meaningful benefits, particularly in infants at a high risk for pulmonary morbidity [17]. One of the most notable studies is the PREMILOC trial (N = 523), which demonstrated that low-dose hydrocortisone, administered within the first 24 h of life and continued for 10 days, significantly improved survival without BPD at 36 weeks PMA (60% vs. 51%, OR 1.48; 95% CI 1.02–2.16; *p* = 0.04) [29]. Importantly, the trial avoided early gastrointestinal perforation by prohibiting the use of prophylactic nonsteroidal anti-inflammatory drugs (NSAIDs) in the first 24 h. In contrast to dexamethasone, hydrocortisone was not associated with increased risks of CP or long-term neurodevelopmental impairment (NDI). In fact, a post hoc analysis of a subgroup of infants born at 24–25 weeks’ gestation showed a significant neurological benefit (moderate-to-severe NDI: 2% in the hydrocortisone group vs. 18% in the placebo group; *p* = 0.02) [29]. The role of hydrocortisone in the later neonatal period has also been evaluated. The STOP-BPD trial (N = 372) assessed high-dose hydrocortisone in ventilator-dependent preterm infants between 7 and 14 days of age. While no significant difference was found in the composite outcome of death or BPD at 36 weeks’ PMA (71% vs. 74%, *p* = 0.54), mortality alone was significantly lower in the hydrocortisone group (15.5% vs. 23.7%, *p* = 0.048) [30]. Similarly to what was observed in DART, hydrocortisone also facilitated extubation at multiple time points (Day 3: 16% vs. 7%, *p* = 0.01; Day 7: 46% vs. 22%, *p* < 0.001; and Day 14: 66% vs. 49%, *p* = 0.001) [30]. At two years corrected age, follow-up data were available for 96% of participants in the study. Among the infants assessed, the combined rate of death or NDI was 56.7% in the hydrocortisone group and 62.7% in the placebo group, with no statistically significant difference (adjusted risk difference −5.2%, 95% CI −15.1% to 4.6%; *p* = 0.28) [31]. Mortality rates were lower in the hydrocortisone group (21.5%) compared to the placebo (29.5%), though this difference did not reach significance (*p* = 0.08). Similarly, the incidence of NDI among survivors was comparable between groups, 43.9% in the hydrocortisone group versus 46.5% in the placebo group (*p* = 0.68), with no notable differences observed in the specific domains of the neurodevelopmental assessment [31]. In a multicenter RCT, Wattenberg et al. [32] enrolled 800 infants of <30 weeks’ gestation who had been intubated for at least 7 days. The infants were randomized to receive hydrocortisone (4 mg/kg/day, tapered over 10 days) or the placebo. The primary efficacy outcome, survival without moderate/severe BPD at 36 weeks PMA, occurred in 16.6% of the hydrocortisone group and 13.2% of the placebo group (adjusted RR 1.27; 95% CI 0.93–1.74). At 22–26 months corrected age, survival without moderate/severe NDI was similar between groups (36.9% vs. 37.3%) [32]. Regarding its safety profile, hydrocortisone was generally well tolerated. In the Wattenberg study, hypertension requiring medication was more common in the hydrocortisone group (4.3% vs. 1.0%), while the incidence of other adverse events was comparable [32]. In contrast to dexamethasone, hydrocortisone has not been associated with an increased risk of cognitive impairment, as seen in data from the EPICE cohort [33]. A recent systematic review and meta-analysis by Doyle et al. [24] reported that hydrocortisone did not significantly reduce the risk of BPD compared to the placebo (RR = 0.98; 95% CI 0.87–1.10), speculating that the predominantly low cumulative doses (<2 mg/kg in most studies) may have been insufficient to elicit a strong anti-inflammatory effect. Similar findings were reported by van de Loo et al. [34], although Doyle did note a mortality benefit with early hydrocortisone use. A very recent meta-regression analysis compared systemic postnatal corticosteroids across 26 RCTs including 3700 infants [35]. The analysis highlighted a differential effect of corticosteroid type: dexamethasone was associated with improved survival, free of CP, in infants with a BPD risk of >70% but was potentially harmful in those with a BPD risk of <30% [35]. In contrast, no substantial benefit or harm was observed with hydrocortisone, suggesting a less pronounced or more context-dependent role. The timing of the treatment did not significantly influence dexamethasone outcomes. Additionally, a meta-analysis by Zhou et al. [36] found that early low-dose hydrocortisone may reduce the combined outcome of BPD or death in infants weighing <1000 g with a prior exposure to chorioamnionitis (OR 0.52; 95% CI 0.32–0.79; NNT = 6), but not in those without such exposure. No significant differences were observed in the individual outcomes of BPD or death regardless of their exposure status [36].

### 2.3. Prednisolone and Methylprednisolone in BPD

Prednisolone and methylprednisolone are synthetic glucocorticoids with similar anti-inflammatory potency and are often used interchangeably in clinical settings [17]. However, their application in the context of BPD remains limited to specific scenarios, and the available evidence for it is still sparse [17]. Unlike dexamethasone or hydrocortisone, these agents have primarily been investigated for late use, once BPD is already established, particularly in infants beyond 36 weeks PMA [17]. Retrospective data have suggested some clinical benefits. In a study by Bhandari et al. [37], prednisolone was administered to infants with established BPD (mean PMA ~38 weeks) who were receiving supplemental oxygen but were not mechanically ventilated. The findings showed that 63% of the treated infants were successfully weaned off supplemental oxygen, indicating a potential role for prednisolone in improving respiratory status. Similarly, Linafelter et al. [38] evaluated a longer course of prednisolone (≥30 days; median duration: 67 days) in infants with severe BPD, including both those on mechanical ventilation and those on noninvasive support. The study noted a significant reduction in respiratory support needs after the first week of treatment. Despite these initial improvements, the benefits appeared to stabilize beyond the first week, and extended therapy did not yield additional clinical gains. Furthermore, prolonged use was associated with notable adverse effects, most significantly impaired linear growth observed by week 4 of the treatment [17]. In Linafelter’s cohort, although a high survival rate to discharge was reported (86%), more than half of the infants (57%) ultimately required a tracheostomy, underscoring the complexity and severity of their condition [38]. Importantly, no prospective RCTs have been conducted to assess the efficacy or safety of prednisolone or methylprednisolone in the prevention or early treatment of BPD [17].

### 2.4. Current Recommendations

A recent position statement from the Canadian Paediatric Society emphasized that the quality of evidence supporting the use of these corticosteroids in established BPD was very low, and therefore, their use could not be routinely recommended [22]. Notably, once BPD has been established, the 2020 European Respiratory Society (ERS) guidelines on the long-term management of children with BPD do not recommend systemic corticosteroids (including dexamethasone or hydrocortisone) [39]. The task force found no strong or direct evidence supporting the benefit of systemic corticosteroids in improving clinically meaningful outcomes in this population. Moreover, concerns regarding the potential adverse effects, including growth suppression and negative neurodevelopmental consequences, contributed to the recommendation against their routine use [39]. As a result, systemic corticosteroids are discouraged in the long-term management of established BPD, particularly as standard therapy. However, in cases of severe disease, persistent symptoms, or frequent hospital readmissions unresponsive to other treatments (e.g., bronchodilators), a carefully monitored trial of corticosteroids may be considered on an individual basis, guided by clinical judgment [39] [Table 1 and Table 2].

## 3. Inhaled Corticosteroids in BPD

Inhaled corticosteroids (ICSs) have been proposed as a strategy to reduce pulmonary inflammation in preterm infants while minimizing systemic exposure. The theoretical advantage lies in delivering the anti-inflammatory effect directly to the lungs, lowering the risk of adverse systemic effects commonly associated with systemic corticosteroids [17]. The current literature includes two distinct approaches: (1) prolonged ICS administration and (2) intratracheal instillation of corticosteroids in combination with a surfactant, typically as a single dose early in life [17].

### 3.1. Budesonide in BPD

Among studies on prolonged ICS use, the NEUROSIS trial is particularly noteworthy as a large, multicentric RCT involving 863 infants born before 28 weeks of gestation [40]. High-dose inhaled budesonide, administered within the first 24 h of life and continued until 32 weeks PMA or weaning off supplemental oxygen, significantly reduced the incidence of BPD compared to the placebo (27.8% vs. 38.0%, *p* = 0.004). However, the composite outcome of death or BPD was only borderline significant (40.0% vs. 46.3%, *p* = 0.05), and mortality alone was numerically higher in the budesonide group (16.9% vs. 13.6%, RR 1.24), although not statistically significant [40]. A long-term two-year follow-up revealed an increased mortality rate in the budesonide group (19.9% vs. 14.5%, RR 1.37, *p* = 0.04), raising concerns about the trade-off between reduced BPD and potential excess mortality. These findings underscore the importance of balancing the pulmonary benefits with overall survival outcomes [40]. A recent Cochrane review supports the notion that the early initiation of ICSs may reduce the risk of BPD and death at 36 weeks’ PMA (RR 0.86, 95% CI 0.75–0.99), with no significant adverse effects reported [35]. The Bassler et al. [40,41] trial, which also evaluated early budesonide use, confirmed a reduction in BPD (27.8% vs. 38.0%; *p* = 0.004) and lower reintubation rates and patent ductus arteriosus (PDA) surgery. However, mortality was again slightly higher in the treatment group, though not statistically significant (16.9% vs. 13.6%). Despite these encouraging findings, ICS delivery in extremely preterm infants remains technically challenging due to low tidal volumes and weak inspiratory efforts, which limit pulmonary deposition [42]. Studies on intratracheal corticosteroid–surfactant administration involve a single or limited number of doses delivered early in life. However, commercial formulations are not yet widely available [42]. Particularly, in one randomized trial involving 265 very low birth-weight neonates, the combined surfactant–budesonide therapy significantly reduced the incidence of death or BPD (42.0% vs. 66.0%; RR 0.58; *p* < 0.001; NNT = 4.1), with no significant differences in their long-term growth or neurodevelopmental outcomes at 2–3 years [43]. A meta-analysis of two RCTs showed that this approach resulted in a 43% reduction in the incidence of BPD (RR 0.57; 95% CI 0.43–0.76; NNT = 5) and a 40% reduction in the composite outcome of death or BPD (RR 0.60; 95% CI 0.49–0.74; NNT = 3), without a statistically significant difference in mortality alone [44]. Furthermore, a recent randomized trial by Liu et al. [45] reinforced these findings, demonstrating that an early intratracheal instillation of budesonide combined with pulmonary surfactant significantly reduced the incidence and severity of BPD, decreased oxygen requirements and the duration of respiratory support, and did not increase glucocorticoid-related complications, supporting the potential safety and efficacy of this combined approach. However, the recently published PLUSS trial investigated the effect of early prophylactic intratracheal budesonide mixed with a surfactant in extremely preterm infants and found no statistically significant improvement in survival that was free of BPD at 36 weeks postmenstrual age, although a slight numerical benefit was observed [46]. Additionally, a meta-analysis including five randomized controlled trials (*n* = 1515) found no significant differences in neurodevelopmental impairment, mortality, or growth parameters between infants who received intratracheal corticosteroids and those who did not (RR for NDI: 0.90; 95% CI: 0.79–1.03; *p* = 0.14), suggesting a neutral safety profile [47].

### 3.2. Other ICSs

Data regarding the use of fluticasone and beclomethasone for the prevention of BPD in preterm infants are currently limited. Both are more commonly used in managing infants with BPD or those who have developed long-term respiratory complications rather than as preventive therapies [24]. A 2024 systematic review provided further clarity by comparing different inhaled corticosteroid regimens. While promising results were highlighted for budesonide, beclomethasone and fluticasone propionate did not demonstrate superior or inferior effects on the clinical outcomes when compared with the controls [48]. While ICSs are thought to present a lower systemic risk profile than systemic corticosteroids, several key limitations prevent their widespread recommendation. First, the quality of evidence is variable, with many studies being small, underpowered, or inconsistent in outcome reporting [49]. Secondly, long-term safety data, especially neurodevelopment-related, remain scarce. However, the existing Cochrane reviews suggest that ICSs initiated after seven days of life do not significantly increase the risk of major adverse events or neurodevelopmental impairments [49,50]. Although evidence ranges from low to very low certainty, no consistent safety concerns such as hypertension, sepsis, or cerebral palsy have emerged, and ICSs may reduce the need for systemic steroid exposure [49,50]. Importantly, the 2022 Cochrane review concluded that the routine early use of ICSs outside of clinical trials is not currently supported. Conversely, the late administration of ICSs appears to offer neither significant benefits nor issues [49]. However, comparative studies between inhaled and systemic corticosteroids are lacking, and no clear evidence exists to support the superiority of one route over the other [49,50].

### 3.3. Current Recommendations

Regarding children with established BPD, the 2020 ERS guidelines do not recommend the use of ICSs in infants beyond 36 weeks PMA or following hospital discharge [39]. This recommendation is based on low-certainty evidence, as the available data do not demonstrate a clear clinical benefit that outweighs the potential risks, particularly the concerns related to growth restriction and adverse neurodevelopmental outcomes with prolonged or repeated exposure. As a result, the routine use of ICSs is discouraged for the long-term management of BPD. However, in select clinical scenarios, such as in cases of severe BPD, persistent respiratory symptoms, or recurrent hospitalizations not responsive to bronchodilators, a carefully monitored, individualized trial of ICSs may be considered [39] [Table 1 and Table 2].

## 4. Conclusions

BPD remains a major challenge in the care of preterm infants, driven by persistent inflammation and impaired lung development. Corticosteroids, both systemic and inhaled, have shown potential in modulating this inflammatory process and OS, but their use must be carefully balanced against potential adverse effects, particularly in terms of neurodevelopment outcomes. Systemic and inhaled corticosteroids continue to play a selective role in BPD management. While dexamethasone and hydrocortisone show potential in specific settings, and budesonide has yielded promising results, particularly when combined with a surfactant, the limitations in long-term safety data and guideline support restrict their routine use. The evidence for other steroid agents remains insufficient. The choice to use corticosteroids for BPD prevention or treatment must be individualized, weighing gestational age, clinical trajectory, and BPD risk. Current evidence underscores the need for larger, high-quality RCTs, particularly those evaluating long-term neurodevelopmental outcomes, biomarker-guided strategies, and optimal timing and dosage regimens to better define the safest and most effective steroid therapies for this vulnerable population.

## Figures and Tables

**Table 1:** Comparison between systemic and inhaled corticosteroids in the prevention and management of bronchopulmonary dysplasia in preterm infants.

	Systemic Corticosteroids	Inhaled Corticosteroids
**Route of administration**	Intravenous or oral	Inhaled (with or without surfactant)
**Main agents**	Dexamethasone, Hydrocortisone, Prednisolone	Budesonide, Beclomethasone, Fluticasone
**Timing of use**	Early (first week), Moderately early (8–14 days), Late (>14 days)	Early initiation (<7 days), some used later
**Primary benefit**	Effective reduction in BPD * incidence, improves extubation	Localized effect with reduced systemic exposure
**Main risks**	Neurodevelopmental impairment, gastrointestinal perforation, infections	Possible increased mortality (early use), technical delivery limitations
**Neurodevelopmental concerns**	Significant with early/high-dose use	Less pronounced or unclear
**Evidence level**	Moderate to High (Cochrane, RCTs)	Low to Moderate (NEUROSIS, Cochrane)
**Use in BPD**	Discouraged (ERS guidelines)	Discouraged (ERS guidelines)

* BPD: bronchopulmonary dysplasia.

**Table 2:** Summary of the timing, duration, main benefits, adverse effects, and current recommendations for systemic and inhaled corticosteroids used in the prevention and management of bronchopulmonary dysplasia (BPD) in preterm infants. This table synthesizes clinical trial evidence and current guidelines to support clinical decision-making.

Steroid	Timing of Initiation	Duration	Main Benefits	Risks	Recommendation	Unresolved Issues
**Dexamethasone**	Early (≤7 days) or Late (>7 days)	3–10 days	Reduced BPD, facilitates extubation	Neurodevelopmental impairment, GI perforation, infections	Use after 1st week in high-risk infants	Ideal timing in moderate-risk infants; long-term safety; biomarker-guided use
**Hydrocortisone**	Early (<24 h) or 7–14 days	10 days	Improved survival without BPD	Hypertension, uncertain NDI effect	Consider in early high-risk or 7–14 d intubated infants	Optimal dose; timing relative to chorioamnionitis; metabolic outcomes
**Prednisolone**	Late (>36 weeks PMA)	≥30 days	Improved O2 weaning in severe BPD	Growth suppression, need for tracheostomy	Limited use in severe, established BPD only	Comparative efficacy; initiation thresholds; endocrine effects
**Budesonide** **(inhaled)**	Early (<24 h)	Until 32 weeks PMA or off O2	Reduced BPD incidence	Possible increased mortality, unclear long-term safety	Not routine; promising with surfactant, more data needed	Long-term safety; neurodevelopmental impact; optimal delivery method

BPD: bronchopulmonary dysplasia; GI: gastrointestinal disease; NDI: neurodevelopmental impairment.

## Abbreviations

The following abbreviations are used in this manuscript:

BPD	Bronchopulmonary dysplasia
CP	Cerebral palsy
ERS	European Respiratory Society
ICS	Inhaled corticosteroids
IL	Interleukin
NDI	Neurodevelopmental impairment
NEC	Necrotizing enterocolitis
NSAIDs	Nonsteroidal anti-inflammatory drugs
OS	Oxidative stress
PDA	Patent Ductus Arteriosus
PMA	Postmenstrual age
ROS	Reactive oxygen species
TNF	Tumor necrosis factor
VILI	Ventilator-induced lung injury
